# Melatonin and Vitamin D Interfere with the Adipogenic Fate of Adipose-Derived Stem Cells

**DOI:** 10.3390/ijms18050981

**Published:** 2017-05-05

**Authors:** Valentina Basoli, Sara Santaniello, Sara Cruciani, Giorgio Carlo Ginesu, Maria Laura Cossu, Alessandro Palmerio Delitala, Pier Andrea Serra, Carlo Ventura, Margherita Maioli

**Affiliations:** 1 Department of Biomedical Sciences, University of Sassari, Viale San Pietro 43/B, 07100 Sassari, Italy; valebasoli@gmail.com (V.B.); sara.santaniello@gmail.com (S.S.); sara.cruciani@outlook.com (S.C.); 2 Laboratory of Molecular Biology and Stem Cell Engineering, National Institute of Biostructures and Biosystems, Via Massarenti, 40138 Bologna, Italy; ventura.vid@gmail.com; 3 Department of Biotechnology, University of Natural Resources and Life Sciences Vienna, Muthgasse 18, A-1190 Vienna, Austria; 4 Clinical and Experimental Medicine Department, University of Sassari, Viale San Pietro 8, 07100 Sassari, Italy; ginesugc@uniss.it (G.C.G.); mlcossu@uniss.it (M.L.C.); paserra@uniss.it (P.A.S.); 5 Azienda Ospedaliero-Universitaria di Sassari, Viale San Pietro 8, 07100 Sassari, Italy; adelitala@tiscali.it; 6 Center for Developmental Biology and Reprogramming (CEDEBIOR), Department of Biomedical Sciences, University of Sassari, Viale San Pietro 43/B, 07100 Sassari, Italy; 7 Stem Wave Institute for Tissue Healing (SWITH), Gruppo VillaMaria and Ettore Sansavini Health Science Foundation, Via Provinciale per Cotignola 9, 48022 Lugo, Ravenna, Italy; 8 Istituto di Ricerca Genetica e Biomedica, Consiglio Nazionale delle Ricerche (CNR), Monserrato, 09042 Cagliari, Italy

**Keywords:** melatonin, vitamin D, stem cells, adipogenesis, cell differentiation, fat depot, adipose tissue, nutraceuticals

## Abstract

Adipose-derived stem cells (ADSCs) represent one of the cellular populations resident in adipose tissue. They can be recruited under certain stimuli and committed to become preadipocytes, and then mature adipocytes. Controlling stem cell differentiation towards the adipogenic phenotype could have a great impact on future drug development aimed at counteracting fat depots. Stem cell commitment can be influenced by different molecules, such as melatonin, which we have previously shown to be an osteogenic inducer. Here, we aimed at evaluating the effects elicited by melatonin, even in the presence of vitamin D, on ADSC adipogenesis assessed in a specific medium. The transcription of specific adipogenesis orchestrating genes, such as *aP2*, peroxisome proliferator-activated receptor γ (*PPAR-γ*), and that of adipocyte-specific genes, including lipoprotein lipase (*LPL*) and acyl-CoA thioesterase 2 (*ACOT2*), was significantly inhibited in cells that had been treated in the presence of melatonin and vitamin D, alone or in combination. Protein content and lipid accumulation confirmed a reduction in adipogenesis in ADSCs that had been grown in adipogenic conditions, but in the presence of melatonin and/or vitamin D. Our findings indicate the role of melatonin and vitamin D in deciding stem cell fate, and disclose novel therapeutic approaches against fat depots.

## 1. Introduction

Adipose tissue represents a widespread component in the human body. It can be found as subcutaneous fat, >80% of the total fat in the body, mainly dealing with energy balance as abdominal, gluteal and femoral depots [1].

Several studies have highlighted visceral fat, which protect internal organs, as a risk factor for many unhealthy conditions such as insulin-resistance, type 2 diabetes mellitus, cardiovascular disease, stroke, and metabolic syndrome [2].

Besides mature adipocytes, fat tissue contains several other cell types, such as stromal-vascular cells (SVC), fibroblasts, smooth muscle cells, pericytes, endothelial cells, and adipogenic progenitor cells or preadipocytes [3].

These adipogenic progenitor cells retain the ability to develop during adulthood, although their development mainly happens in early postnatal life [3,4,5,6]. In this regard, adipose tissue contains adipose precursor cells, called preadipocytes, which arise from mesenchymal stem cells (MSCs) resident in a particular tissue zone called the “niche” [7,8], a specific environment defined at the nanoscale level which fashions topographies that can either promote or erase pluripotency of the embedded cells [5].

Adipocyte differentiation is characterized by the activation of specific adipogenic genes and fine epigenetic regulation [9]. These early markers of commitment include the transcription factor CCAAT/enhancer binding protein (C/EBP) gene family and peroxisome proliferator-activated receptor γ (*PPAR-γ*) [10], which encode an adipocyte-specific nuclear hormone receptor. These marker genes, in turn, activate the adipocyte-specific *aP2* (adipocyte fatty acid binding protein 2) gene, coding for a carrier protein for fatty acids [11] and lipoprotein lipase (LPL), another important player involved in fatty acid metabolism, thus orchestrating the expression of intermediate and late adipose phenotype-markers as well as triglyceride accumulation [5,9].

Recent findings highlight the role of vitamin D and its active form 1,25(OH)2D in influencing the gene expression of key molecules involved in adipogenesis [12], fat oxidation and lipolysis by the activation of the nicotinamide adenine dinucleotide (NAD)-dependent sirtuin1 pathway [13]. Vitamin D3 has a range of physiological functions in the body, the most important being the maintenance of the calcium and phosphorus balance [14] in osteoblasts, where it usually inhibits cellular proliferation and induces differentiation [15].

Through this way, vitamin D is able to affect MSC differentiation towards the osteogenic phenotype [16], contributing to the fine regulation of adipogenic towards osteogenic balancing [17]. Vitamin D and vitamin D receptor (VDR) are expressed in adipocytes, and regulate adipogenic gene expression and apoptosis [18]. The deficiency of vitamin D and VDR has been proposed as a cause of obesity [19]. Moreover, VDR and hydrolases have been recently found in porcine ADSCs, indicating that these cells are able to respond to and metabolize vitamin D [20].

Alongside vitamin D, melatonin, besides being a neurohormone synthesized by the pineal gland, controlling many functions such as circadian rhythms and immune response, has also been recently found to be involved in the osteogenic differentiation of MSCs [21,22]. It is also known to influence adipocyte metabolism [23], resulting in changes in body weight [24]. Recent findings indicate the role of melatonin in anti-inflammatory processes, in which it promotes adipocyte pyroptosis, and these findings highlighted a future therapeutic role of melatonin in inflammatory-related obesity [25].

Within this context, we have recently unraveled the role of melatonin, together with other compounds, in the osteogenic differentiation of stem cells derived from human dental pulp [26]. In the present study, we aimed to evaluate the effects elicited by melatonin, alone or in combination with vitamin D, on the adipogenic commitment of adipose-derived mesenchymal stem cells (ADSCs) cultured in a conditioned medium specific for the establishment of the adipogenic lineage. We hypothesized that melatonin together with vitamin D, even in a strong adipogenic environment, may be able to inhibit adipogenic differentiation in cells that seem to maintain an “adipogenic memory” [27]. Our findings could add interesting molecular insights in stem cell differentiation, and open up novel strategies for future therapeutic approaches for fighting fat depots.

## 2. Results

### 2.1. Melatonin Together with Vitamin D Counteract Adipose-Derived Mesenchymal Stem Cell (ADSC) Expression of Key Adipogenic Genes

**Figure 1:**
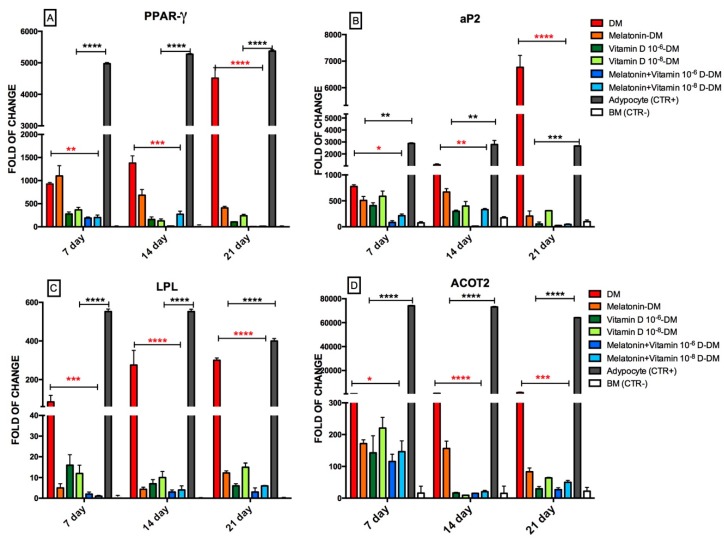
Effect of melatonin and vitamin D exposure on the expression of genes orchestrating ADSC adipose commitment. Cells were exposed for 7, 14, or 21 days in the presence of Differentiation Medium (DM), or Differentiation Medium together with melatonin (Melatonin-DM), different concentrations of vitamin D3 (Vitamin D 10^−6^ M-DM or Vitamin D 10^−8^ M-DM), or both (Melatonin + Vitamin D 10^−6^ M-DM or Melatonin + Vitamin D 10^−8^ M-DM). The amounts of PPAR γ (**A**), aP2 (**B**) LPL (**C**) and ACOT2 (**D**) mRNA were normalized to Glyceraldehyde-3-Phosphate-Dehidrogenase (*GAPDH*). The mRNA expression of cells exposed to Differentiation Medium plus melatonin (Melatonin-DM, orange bars), or to Differentiation Medium plus vitamin D (Vitamin D 10^−6^ M-DM, dark green bars, or Vitamin D 10^−8^ M-DM, light green bars) or to both melatonin and vitamin D together with the Differentiation Medium (Melatonin + Vitamin D 10^−6^ M-DM, dark blue bars or Melatonin + Vitamin D 10^−8^ M-DM, light blue bars) was plotted at each time point as a fold of change relative to the expression of the corresponding gene in the control of undifferentiated cells defined as 1 (mean ± SD; *n* = 6). All data from Melatonin-DM, Vitamin D 10^−6^ M-DM, or Vitamin D 10^−8^ M-DM, or from Melatonin + Vitamin D 10^−6^ M-DM or Melatonin + Vitamin D 10^−8^ M-DM at each time point were significantly different from those in cells exposed to the Differentiation Medium alone (DM, red bars). Grey bars represent adipocytes positive controls, white bars (BM) represent negative controls. mRNA levels from cells that had been exposed to melatonin and vitamin D at different concentrations and combinations were significantly different from each other at each time point. Data are expressed as mean ± SD (* *p* ≤ 0.05, ** *p* ≤ 0.01, *** *p* ≤ 0.001, **** *p* ≤ 0.0001). Significant difference from the DM is marked by red asterisks; significant difference from the adipocytes positive control is marked by black asterisks. All significant differences from the BM control are not shown.

Figure 1 shows the expression of genes usually upregulated during the adipogenic commitment of ADSCs. In particular, as expected, *PPAR-γ* gene expression, a main orchestrator of adipogenic commitment, was upregulated in ADSCs exposed to an adipogenic medium for 7, 14 or 21 days (Figure 1A). On the other hand, the gene expression of *PPAR-γ* was significantly counteracted by the addition of vitamin D or melatonin, alone or in combination, to the adipogenic medium. This effect was particularly evident in ADSCs that had been concomitantly exposed to vitamin D (10^−6^ M) and melatonin. The same figure shows the effect of the different conditioned media on the gene expression of adipocyte fatty acid binding protein, *aP2*, in ADSCs cultured under different conditioned media (Figure 1B). As expected, this gene was actively expressed during adipogenic differentiation induced by culturing cells in differentiation medium (DM), while under the other experimental conditions tested *aP2* gene expression was significantly inhibited, being only faintly detectable when ADSCs were committed towards the adipogenic phenotype in the presence of both vitamin D (10^−6^ and 10^−8^ M) and melatonin.

**Figure 2:**
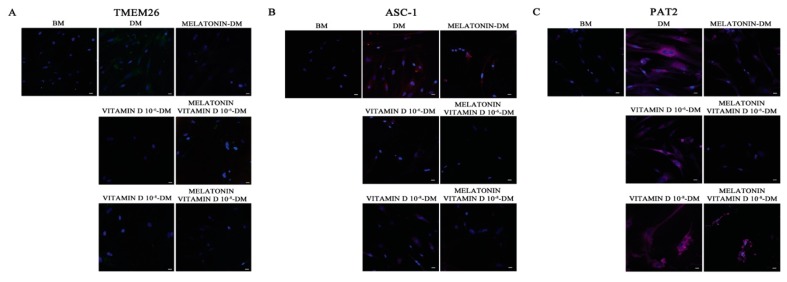
Analysis of adipogenic specific proteins. Immunohistochemical analysis of the expression of TMEM 16 (**A**), ASC-1 (**B**) and PAT2 (**C**) was assessed in cells cultured for 21 days in basic medium (BM) as a negative control and in the presence of the Differentiation Medium (DM) or the Differentiation Medium together with melatonin (Melatonin-DM) or with 10^−6^ vitamin D (Vitamin D 10^−6^ M-DM) or with 10^−8^ M vitamin D (Vitamin D 10^−8^ M-DM), or with both melatonin and vitamin D (Melatonin + Vitamin D 10^−6^ M-DM, Melatonin + Vitamin D 10^−8^ M-DM). Nuclei are labelled with 4,6-diamidino-2-phenylindole (DAPI, blue). Scale bars: 40 µm. The figures are representative of five separate experiments. For each differentiation marker, fields with the highest yield of positively stained cells are shown.

Similar results were obtained evaluating the expression of the later adipogenic-related genes, Lipoprotein lipase (*LPL*) and acyl-CoA thioesterase 2 (*ACOT2*) (Figure 1C,D). In particular, *LPL* gene expression was induced in ADSCs cultured in an adipogenic medium after 7 days in culture, while it was significantly inhibited in the presence of melatonin, vitamin D (10^−6^ or 10^−8^ M), or when these compounds were contemporarily added to the cultures (Figure 2C). Similarly, *ACOT2* transcription, while being induced after a 7-day culture in an adipogenic medium, was markedly downregulated by the presence of melatonin or vitamin D (10^−6^ or 10^−8^ M), with a stronger inhibitory action observed upon their concomitant administration to the culture medium (Figure 1D).

### 2.2. Vitamin D and Melatonin Affect the Appearance of Adipogenic Specific Proteins

Figure 2 shows the immunofluorescence analysis of the adipogenic phenotype-marker proteins TMEM26, ASC-1, and PAT2. All these proteins are clearly evident in ADSCs committed to the adipogenic phenotype in the presence of the Differentiation Medium (DM, Figure 2A–C). On the other hand, TMEM26, ASC-1, and PAT2 were only faintly detectable in ADSCs committed towards adipogenesis in the presence of melatonin or vitamin D (10^−6^ or 10^−8^ M) (Figure 2, Melatonin-DM, Vitamin D 10^−8^ M-DM, Vitamin D 10^−6^ M-DM). Interestingly, when these compounds were contemporarily present in the Differentiation Medium, TMEM26, and ASC-1 were completely undetectable (Figure 2, Melatonin + Vitamin D 10^−6^ M-DM, Melatonin + Vitamin D 10^−8^ M-DM). PAT2 was inhibited by both melatonin and vitamin D at different concentrations, although still faintly detectable (Figure 2C).

### 2.3. Melatonin and Vitamin D Inhibit Intracellular Lipid Accumulationin in ADSCs

**Figure 3:**
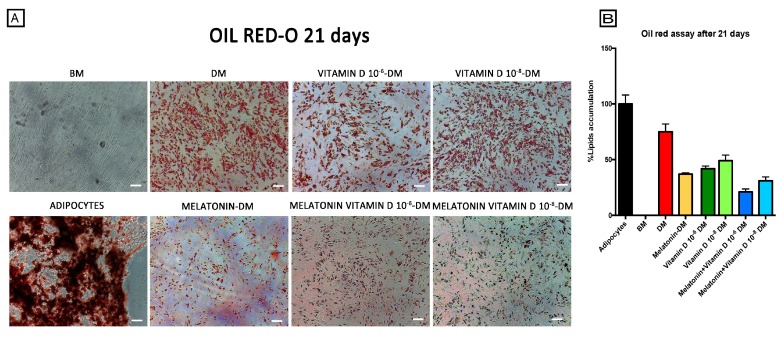
Effect of melatonin and vitamin D exposure on lipid accumulation in ADSCs during adipose differentiation. (**A**) shows the oil red lipid accumulation in mature adipocytes (positive control), adipose-derived stem cells cultured in basic medium (BM), and adipose-derived stem cells exposed for 21 days in the presence of the Differentiation Medium or the Differentiation Medium together with melatonin (Melatonin-DM) or with 10^−6^ vitamin D (Vitamin D 10^−6^ M-DM) or with 10^−8^ M vitamin D (Vitamin D 10^−8^ M-DM), or with both melatonin and vitamin D (Melatonin + Vitamin D 10^−6^ M-DM, Melatonin + Vitamin D 10^−8^ M-DM), scale bar 100 μm. The amount of lipid accumulation and (**B**) was calculated using ImageJ, with mature adipocytes as a positive control (black bar) and adipose-derived stem cells cultured in basic medium (white bar) as negative control for lipid accumulation. Adipose-derived stem cells were exposed for 21 days in the presence of Differentiation Medium (red bar) or Differentiation Medium together with melatonin (orange bar) or with 10^−6^ M vitamin D (Vitamin D 10^−6^ M-DM, dark green bar) or with 10^−8^ M vitamin D (Vitamin D 10^−8^ M-DM, light green bar) or with both (Melatonin + Vitamin D 10^−6^ M-DM, dark blue bar, Melatonin + Vitamin D 10^−8^ M-DM, light blue bar). Data are expressed as mean ± SD.

Consistent with the observations from gene expression and immunohistochemical analyses, we found that during the adipogenic commitment in ADSCs exposed to the Differentiation Medium, the presence of melatonin alone (Figure 3, Melatonin-DM) counteracted intracellular lipid accumulation. Similarly, the use of vitamin D3 at different concentrations (Figure 3, Vitamin D 10^−8^ M-DM and Vitamin D 10^−6^ M-DM) was able to inhibit the adipogenic differentiation and fat droplet formation, as compared to adipocytes, or to ADSCs cultured in Differentiation Medium alone (Figure 3, DM). The inhibition of adipogenic differentiation was higher when melatonin and vitamin D were added simultaneously to the Differentiation Medium (Figure 3, Melatonin + Vitamin D 10^−8^ M-DM and Melatonin + Vitamin D 10^−6^ M-DM).

## 3. Discussion

Recent findings highlighted a novel feature of adipose tissue, which is now thought to be routinely fed by precommitted cells known as preadipocytes [4,5,6,28]. These cells belong to the stem cell niche [7], a specific environment which can strongly influence the behaviour of ADSCs. Recently published results suggest that ADSCs can retrieve a sort of memory capable of influencing their future behaviour [29]. Along this pathway, obesity could be read as a result of dysfunctional adipocyte features, involving not only their size, but also their number and deeply related they are to the differentiating processes affecting stem cell-derived preadipocytes [30]. Understanding how these precommitted cells or even their ancestors decide this fate could disclose novel natural molecules and/or lead to the development of synthetic agents capable of affecting this phenotypic decision, thus counteracting adipose tissue depots and obesity [11]. Recently, melatonin emerged not only as a molecule involved in different neuroendocrine processes, but also as an orchestrator of different cellular fates, influencing MSC commitment towards selected lineages, such as osteogenic, chondrogenic, adipogenic, and myogenic phenotypes, by recruiting the Wnt/β-catenin pathway, MAPKs and TGF-β signaling [31]. In this regard, we have recently demonstrated the role of melatonin, together with other molecules, in orchestrating osteogenesis in human dental pulp stem cells [26]. Vitamin D was also recently found to be a molecule capable of inducing stem cell commitment towards the osteogenic phenotype [22].

Here, we expanded our understanding of the biological actions of melatonin and vitamin D, showing that these molecules are able to exert an inhibition of the adipogenic process. The effect was clearly evident even in the presence of a strong driving force towards this lineage, such as the cell growth in a specific Differentiation Medium. The finding that melatonin and vitamin D could be used to promote the synergistic inhibition of adipogenesis, with higher inhibitory action compared to the effects elicited by each individual molecule, suggests that the downregulation of fat accumulation may be tuned to achieve a fine grading of depot accumulation. Worthy to note, such negative regulation of adipogenesis occurred at different interconnected levels, involving the transcription of genes responsible for early commitment, the expression of adipogenic-restricted marker proteins at the intact cell level, and the terminal phenotypic specification. These findings, although in vitro, may pave the way to individualized approaches of precision medicine aiming at decreasing pathological fat accumulation.

## 4. Materials and Methods

### 4.1. Cell Isolation and Maintenance

Subcutaneous adipose tissue was harvested during surgery processes for different reasons, from human adult patients; males (*n* = 4) and females (*n* = 8) (*n* = 12, age = 45 ± 15 years, BMI: 22 ± 3 kg/m^2^).

The patients gave written informed consent according to the approval of this study by the Ethics Committee Review Boards for Human Studies in Sassari (n° ETIC 240I/CE 26 July 2016, Ethical committee, ASL Sassari).

Isolation procedures of fat tissue components are convergent in the standard methods, based on enzymatic digestion. Those methods are designed to separate the two easily recognizable fractions, mature adipocytes and the stromal vascular fraction (SVF). The composition of the SVF is commonly known, however, to include preadipocytes, endothelial cells, pericytes, fibroblasts, adipose-derived stem cells (ADSCs) and hematopoietic stem cells. After collagenase digestion, mature adipocytes with a high fat content are separated as a floating layer. All cells which remain after the removal of mature adipocytes constitute the SVF [32].

Briefly, 2 g of subcutaneous adipose tissue were washed repeatedly with sterile Dulbecco’s phosphate buffered saline (DPBS) (Euroclone, Milano, Italy) containing 200 U/mL penicillin and 0.1 mg/mL streptomycin (Euroclone, Milano, Italy), in order to remove the blood cells. They were then dissociated into small fragments with a scalpel, followed by enzymatic digestion with 0.1% Type I collagenase (Gibco Life Technologies, Grand Island, NY, USA) for 60 min at 37 °C in Hanks balanced salt solution (HBSS; Sigma Aldrich Chemie GmbH, Munich, Germany) under continuous, gentle agitation.

After neutralization of the enzyme with 10% foetal bovine serum (FBS) (Life Technologies, Grand Island, NY, USA) and filtering (70 μm cell strainer) (Euroclone, Milano, Italy), samples were centrifuged at 600× *g* for 10 min to separate distinct cell fractions. The obtained supernatant was composed of mature adipocytes and the pellet fraction consisted of the SVF components, in which the ADSCs were presumably present. The adipocytes were transferred into a basic medium (BM), Dulbecco’s modified Eagle’s Medium (DMEM) (Life Technologies Grand Island, NY, USA) supplemented with 10% foetal bovine serum (FBS) (Life Technologies, Grand Island, NY, USA), 200 mM l-glutamine (Euroclone, Italy), and 200 U/mL penicillin—0.1 mg/mL streptomycin (Euroclone, Milano, Italy), and plated in 12 mL flasks filled with this medium. The flasks were placed in an upside-down fashion in the culture incubator at 37 °C with 5% CO_2_ and saturated humidity for 10–14 days. After the mature adipocytes adhered to ceiling plane, the medium was aspirated and the bottle was placed in an upside-down fashion [33].

The SVF cells were resuspended in basic medium (BM), plated in 12 cm^2^ flasks for culturing and transferred at 37 °C to an incubator with 5% CO_2_ and saturated humidity.

After 48 h of incubation, the cultures were washed with DPBS and kept in the fresh medium. The culture medium was changed every 3 days. When the cells reached 80–90% confluence, they were harvested using 0.25% Trypsin EDTA (Euroclone, Milano, Italy), counted and passaged into new flasks.

### 4.2. Identification of ADSCs after Culture

The positive selection of adipose stem cells from the stromal vascular fraction (SVF) isolated from adipose tissue was obtained with a primary monoclonal anti-c/kit (CD117) antibody (Miltenyi Biotec, Minneapolis, MN, USA) and, subsequently, cell suspensions were magnetically labelled in the columns with a secondary antibody directly conjugated to MicroBeads (MACS Miltenyi Biotec, Bologna, Italy).

### 4.3. Characterization of Adipose-Derived Stem Cells

Flow cytometry analysis was exploited to assess the percentage of mesenchymal markers and the homogeneity of the isolated population. After fixation using 1% formaldehyde for 10 min at room temperature, cells were permeabilized using a permeabilization buffer (eBioscienceMilano, Italy) for 30 min at 4 °C. After a washing step, cells were incubated with primary antibodies directed against CD73, CD90 (BD Biosciences, San Jose, CA, USA), CD105 (Santa Cruz Biotechnology, Heidelberg, Germany), CD45 and CD31 (Sigma-Aldrich, Munich, Germany) (all at 1 µg/10^6^ cells) for 1 h at 4 °C, and with 1 µg of fluorescein isothiocyanate (FITC)-conjugated secondary antibody for 1 h at 4 °C in the dark. After washing, cells were analysed on a flow cytometer (CytoFlex, Backman Coulter, Milan, Italy) by collecting 10,000 events.

### 4.4. Adipose-Derived Stem Cells Culturing

After immunoselection, adipose-derived stem cells (ADSCs) were grown in a basic medium (BM). ADSCs were committed towards the adipogenic phenotype by switching to a conditioned Differentiation Medium (DM), containing Dulbecco’s modified Eagle’s Medium (DMEM, Life Technologies USA) and supplemented with 10% foetal bovine serum (FBS Life Technologies USA), 200 mM l-glutamine (Euroclone, Italy), 200 U/mL penicillin−0.1 mg/mL streptomycin (Euroclone, Milano, Italy), 1 µM dexamethasone, 0.5 µM hydrocortisone, 60 µM indomethacine, and 0.5 mM 3-isobutyl-1-methylxanthine (IBMX Sigma Aldrich Chemie GmbH, Munich, Germany). Other groups of cells were exposed to the DM in the presence of melatonin (0.01 M) (Melatonin-DM), or 10^−8^ M vitamin D (Vitamin D10^−8^ M-DM) or 10^−6^ M vitamin D (Vitamin D 10^−6^ M-DM) or melatonin plus 10^−6^ M mitamin D (Melatonin + Vitamin D 10^−6^ M-DM), or melatonin plus 10^−8^ M vitamin D (Melatonin + Vitamin D10^−8^ M-DM). These concentrations were derived from previously published data (for melatonin [26]) and from experiments in which the lower concentration of Vitamin D proved to be inefficient, while the higher concentration proved to be toxic (data not shown).

### 4.5. RNA Extraction and Quantitative Polymerase Chain Reaction

RNA from undifferentiated ADSCs (BM) at passage 5, as well as adipocytes, were isolated at days 0, and at day 7, 14 and 21 from ADSCs committed to the adipogenic phenotype in the presence of the previously described conditions. These isolated components were used for quantitative polymerase chain reaction. RNA was collected by means of the ChargeSwitch total RNA Cell Kits (Life Technologies, Grand Island, NY, USA). Approximately 1 µg of total RNA was reverse-transcribed into cDNA using the Superscript Vilo cDNA synthesis kit (Life Technologies USA), according to the manufacturer’s protocol.

Quantitative polymerase chain reaction was performed using a CFX Thermal Cycler (Bio-Rad) in triplicate (Applied Biosystems), and then incubated under standard qRT-PCR conditions (50 °C for 2 min, 95 °C for 2 min, and then cycled at 95 °C for 15 s, 55–59 °C for 30 s and 60 °C for 1 min, for 40 cycles), according to the qRT-PCR protocol specified in the Platinum^®^ Quantitative PCR SuperMix-UDG Kit.

A 2× SuperMix whit SYBR Green I, 0.1 µM of each primer, and 3 µL cDNA generated from 1 μg of the total RNA template were mixed in 25 µL volumes and added to each reaction. Target Ct values were normalized to *GAPDH*, considered as a reference gene, while the mRNA levels of ADSCs treated with the different conditioned media and mature adipocytes were expressed as fold of change (2^−∆∆*C*t^) relative to the mRNA levels observed at passage 5 when ADSCs reached 80% confluence before starting treatment. Each experiment included a distilled water control.

The qRT-PCR analysis was performed for the following set of genes: adipocyte fatty acid binding protein (*aP2*), peroxisome proliferator-activated receptor gamma (*PPAR-γ*), lipoprotein lipase (*LPL*), and acyl-CoA thioesterase 2 (*ACOT2*).

All primers used (Invitrogen) in this study were spanning exons and were from Invitrogen. Specific primers for *GAPDH, aP2*, *PPAR-γ* have been previously described [34], while specific primers for *LPL* and *ACOT2* were designed using “PRIMER BLAST” and “primer3,” all of which are described in Table 1.

### 4.6. Immunostaining

ADSCs were cultured for 21 days in a differentiation medium in the presence or not of melatonin or vitamin D3, or both, while control cells were maintained undifferentiated in the presence of a basic medium.

After 21 days, the cells were detached with 0.25% Trypsin EDTA (Euroclone, Italy) and the resulting cell suspension was cultured in 8-well chamber slides (BD-falcon) at low density to allow for the visualization of individual cells. Cells were then fixed with 100% Methanol (sigma Aldrich Chemie GmbH, Germany) at −20 °C for 30 min and then at −80 °C for 30 min. After permeabilization by 0.1% Triton X-100 (Life Technologies, USA)-PBS, cells were washed in PBS three times for 5 min and incubated with 3% Bovine Serum Albumine (BSA)—0.1% Triton X-100 in PBS (Life Technologies, USA) for 30 min and then exposed overnight at 4 °C to the primary anti-mouse monoclonal antibodies directed against activating signal cointegrator-1 (ASC-1) (Santa Cruz Biotechnology, Heidelberg, Germany), proton-coupled amino acid transporter (PAT2) (Santa Cruz Biotechnology), and transmembrane protein 26 (TMEM26) (Abcam, Cambridge, UK).

Finally, cells were washed in PBS two times for 5 min and stained at 37 °C for 1 h in the dark with the fluorescence-conjugated goat anti rabbit IgG secondary antibody or TRITC-conjugated anti mouse. All microscopy analyses were performed with a confocal microscope (TCS SP5, Leica, Nussloch, Germany). DNA was visualized with 1 µg/mL 4,6-diamidino-2-phenylindole (DAPI).

### 4.7. Red Oil Asssay

Cells were cultured for 21 days on tissue cultured in 24 wells (BD-falcon) in Differentiation Medium in the presence or not of melatonin or vitamin D3, or both of them. After 21 days, cells were fixed for 30 min at RT in 10% formalin, then washed twice in H_2_O and in 60% isopropanol for 5 min. Cells were stained for 15 min in oil red solution, washed once with H_2_O and counterstained for 2 min in Mayer’s hematoxilin solution. Cells were then washed and adipogenesis was evaluated by light microscopy. The analysis of lipid accumulation was performed using ImageJ, using adipocyte cells as a positive control.

### 4.8. Statistical Analysis

Statistical analysis was performed using Statistical Package for the Social Sciences version 13 software (SPSS Inc., Chicago, IL, USA); this software gives a corrected *p*-value for multiple comparisons. For this study, the non-parametric Kruskal-Wallis rank sum and Wilcoxon signed-rank test were applied. The tests and results with *p* < 0.05 were considered statistically significant. The former was used to evaluate the distributions and homogeneity of each group variance at different times of observation; the latter was used to evaluate, in the same group, differences between the data collected over a period of observation and the reference value in adipogenic conditions.

## Figures and Tables

**Table 1:** Primers sequences.

Primer Name	Forward	Reverse
***GAPDH***	GAGTCAACGGATTTGGTCGT	GACAAGCTTCCCGTTCTCAG
***aP2***	AGACATTCTACGGGCAGCAC	TCATTTTCCCACTCCAGCCC
***PPAR-γ***	AATCCGTCTTCATCCACAGG	GTGAAGACCAGCCTCTTTGC
***LPL***	CAGGATGTGGCCCGGTTTAT	GGGACCCTCTGGTGAATGTG
***ACOT2***	GAGGTCTTCACACTGCACCA	TCTTGGCCTCGAATGGTATC

## References

[1] Shen W., Wang Z.M., Punyanita M., Lei J., Sinav A., Kral J.G., Imielinska C., Ross R., Heymsfield S.B. (2003). Adipose tissue quantification by imaging methods: A proposed classification. Obes. Res..

[2] Finelli C., Sommella L., Gioia S., la Sala N., Tarantino G. (2013). Should visceral fat be reduced to increase longevity?. Ageing Res. Rev..

[3] Vasan S.K., Karpe F. (2016). Adipose tissue: Fat, yet fit. Nat. Rev. Endocrinol..

[4] Poulos S.P., Hausman D.B., Hausman G.J. (2010). The development and endocrine functions of adipose tissue. Mol. Cell. Endocrinol..

[5] Rosen E.D., MacDougald O.A. (2006). Adipocyte differentiation from the inside out. Nat. Rev. Mol. Cell Biol..

[6] Moreno-Navarrete J.M., Fernández-Real J.M., Symonds M.E. (2012). Adipose Tissue Biology.

[7] Augello A., de Bari C. (2010). The regulation of differentiation in mesenchymal stem cells. Hum. Gene Ther..

[8] Poulsom R., Alison M.R., Forbes S.J., Wright N.A. (2002). Adult stem cell plasticity. J. Pathol..

[9] Li H., Xiao L., Wang C., Gao J., Zhai Y. (2010). Epigenetic regulation of adipocyte differentiation and adipogenesis. J. Zhejiang Univ. Sci. B.

[10] Yang H.N., Park J.S., Woo D.G., Jeon S.Y., Do H.J., Lim H.Y., Kim J.H., Park K.H. (2011). C/EBP-α and C/EBP-β-mediated adipogenesis of human mesenchymal stem cells (hMSCs) using PLGA nanoparticles complexed with poly(ethyleneimmine). Biomaterials.

[11] Moseti D., Regassa A., Kim W.K. (2016). Molecular regulation of adipogenesis and potential anti-adipogenic bioactive molecules. Int. J. Mol. Sci..

[12] Wood R.J. (2008). Vitamin D and adipogenesis: New molecular insights. Nutr. Rev..

[13] Chang E., Kim Y. (2016). Vitamin D decreases adipocyte lipid storage and increases NAD-SIRT1 pathway in 3T3-L1 adipocytes. Nutrition.

[14] Dusso A.S., Brown A.J., Slatopolsky E. (2005). Vitamin D. Am. J. Physiol. Ren. Physiol..

[15] Kawase T., Oguro A. (2004). Granulocyte colony-stimulating factor synergistically augments 1,25-dihydroxyvitamin D3-induced monocytic differentiation in murine bone marrow cell cultures. Horm. Metab. Res..

[16] Song I., Kim B.S., Kim C.S., Im G. (2011). Effects of BMP-2 and vitamin D3 on the osteogenic differentiation of adipose stem cells. Biochem. Biophys. Res. Commun..

[17] Hoshiba T., Kawazoe N., Chen G. (2012). The balance of osteogenic and adipogenic differentiation in human mesenchymal stem cells by matrices that mimic stepwise tissue development. Biomaterials.

[18] Abbas M.A. (2017). Physiological functions of Vitamin D in adipose tissue. J. Steroid Biochem. Mol. Biol..

[19] Chang E., Kim Y. (2017). Vitamin D insufficiency exacerbates adipose tissue macrophage infiltration and decreases AMPK/SIRT1 activity in obese rats. Nutrients.

[20] Valle Y.L., Almalki S.G., Agrawal D.K. (2016). Vitamin D machinery and metabolism in porcine adipose-derived mesenchymal stem cells. Stem Cell Res. Ther..

[21] Radio N.M., Doctor J.S., Witt-Enderby P.A. (2006). Melatonin enhances alkaline phosphatase activity in differentiating human adult mesenchymal stem cells grown in osteogenic medium via MT2 melatonin receptors and the MEK/ERK (1/2) signaling cascade. J. Pineal Res..

[22] Kato H., Ochiai-Shino H., Onodera S., Saito A., Shibahara T., Azuma T. (2015). Promoting effect of 1,25(OH)2 vitamin D3 in osteogenic differentiation from induced pluripotent stem cells to osteocyte-like cells. Open Biol..

[23] Sanchez-Hidalgo M., Lu Z., Tan D., Maldonado M.D., Reiter R.J., Gregerman R.I. (2007). Melatonin inhibits fatty acid-induced triglyceride accumulation in ROS17/2.8 cells: Implications for osteoblast differentiation and osteoporosis. Am. J. Physiol. Regul. Integr. Comp. Physiol..

[24] Liu Z., Gan L., Luo D., Sun C. (2017). Melatonin promotes circadian rhythm-induced proliferation through Clock/histone deacetylase 3/c-Myc interaction in mouse adipose tissue. J. Pineal Res..

[25] Liu Z., Gan L., Xu Y., Luo D., Ren Q., Wu S., Sun C. (2017). Melatonin alleviates inflammasome-induced pyroptosis through inhibiting NF-κB/GSDMD signal in mice adipose tissue. J. Pineal Res..

[26] Maioli M., Basoli V., Santaniello S., Cruciani S., Delitala A.P., Pinna R., Milia E., Grillari-Voglauer R., Fontani V., Rinaldi S. (2016). Osteogenesis from Dental Pulp Derived Stem Cells: A Novel Conditioned Medium Including Melatonin within a Mixture of Hyaluronic, Butyric, and Retinoic Acids. Stem Cells Int..

[27] Chatterjee T.K., Basford J.E., Yiew K.H., Stepp D.W., Hui D.Y., Weintraub N.L. (2014). Role of histone deacetylase 9 in regulating adipogenic differentiation and high fat diet-induced metabolic disease. Adipocyte.

[28] Lowe C.E., O’Rahilly S., Rochford J.J. (2011). Adipogenesis at a glance. J. Cell Sci..

[29] Kim W.-S., Han J., Hwang S.-J., Sung J.-H. (2014). An update on niche composition, signaling and functional regulation of the adipose-derived stem cells. Expert Opin. Biol. Ther..

[30] Cawthorn W.P., Scheller E.L., MacDougald O.A. (2012). Adipose tissue stem cells meet preadipocyte commitment: Going back to the future. J. Lipid Res..

[31] Luchetti F., Canonico B., Bartolini D., Arcangeletti M., Ciffolilli S., Murdolo G., Piroddi M., Papa S., Reiter R.J., Galli F. (2014). Melatonin regulates mesenchymal stem cell differentiation: A review. J. Pineal Res..

[32] Toda S., Uchihashi K., Aoki S., Sonoda E., Yamasaki F., Piao M., Ootani A., Yonemitsu N., Sugihara H. (2009). Adipose tissue-organotypic culture system as a promising model for studying adipose tissue biology and regeneration. Organogenesis.

[33] Zhang H.H., Kumar S., Barnett A.H., Eggo M.C. (2000). Ceiling culture of mature human adipocytes: Use in studies of adipocyte functions. J. Endocrinol..

[34] Maioli M., Rinaldi S., Santaniello S., Castagna A., Pigliaru G., Delitala A., Bianchi F., Tremolada C., Fontani V., Ventura C. (2013). Radio Electric Asymmetric Conveyed Fields and Human Adipose-Derived Stem Cells Obtained With a Non-Enzymatic Method and Device: A Novel Approach To Multipotency. Cell Transplant..

